# Consciousness Without Content: A Look at Evidence and Prospects

**DOI:** 10.3389/fpsyg.2020.01992

**Published:** 2020-08-07

**Authors:** Narayanan Srinivasan

**Affiliations:** ^1^Centre of Behavioural and Cognitive Sciences, University of Allahabad, Allahabad, India; ^2^Interdisciplinary Program in Cognitive Science, Indian Institute of Technology Kanpur, Kanpur, India

**Keywords:** consciousness, *Turiya* state, continuity of conscious experience, dreamless sleep, minimal phenomenal experience

## Abstract

Many traditions in the East have proposed that consciousness without content is possible and could be achieved with mental training. However, it is not clear whether such a state is possible given that intentionality is a critical property of mentality and consciousness in many theories of consciousness. A prominent recent attempt to account for such states of “minimal phenomenal experience” is the ascending reticular arousal system (ARAS) model, which proposes a specific type of non-conceptual representational content to address such a state. Consciousness without content can also be understood by studying related or similar states of minimal phenomenal experience and this paper discusses such findings from such states including dreamless sleep experience and their implications. One way to argue for the need for proposing consciousness without content is to locate a property of consciousness that would necessitate postulating it. A continuous state of consciousness without content may be needed to understand continuity of conscious experience. Finally, I discuss the implications of consciousness without content for current theories of consciousness.

## Introduction

Multiple contemplative traditions report exceptional experiences and these experiences pose critical questions for the study of consciousness (Thompson, 2014; Metzinger, 2019). These exceptional experiences have been used to characterize and define states of consciousness. One of the earliest writings on states of consciousness comes from the Upanishads (Olivelle, 1998; Thompson, 2014). Verse 7 of the Mandukya Upanishad mentions four states of consciousness. Three states of consciousness are familiar, which are wakefulness, dreaming, and sleep. The most interesting and unusual is the fourth state called *Turiya*. *Turiya* is defined as follows: “They consider the fourth quarter as perceiving neither what is inside nor what is outside, nor even both together; not as a mass of perception, neither as perceiving nor as not perceiving; as unseen; as beyond the reach of ordinary transaction; as ungraspable; as without distinguishing marks; as unthinkable; as indescribable; as one whose essence is the perception of itself alone; as the cessation of the visible world; as tranquil; as auspicious; as without a second. That is the self (atman), and it is that which should be perceived (Olivelle, 1998, p. 475).” *Turiya* is also mentioned in other Upanishads as well; for example, in Brihadaranyaka Upanishad, chapter 5.14.3 (Olivelle, 1998). *Turiya* is not simply another state of consciousness but is considered the basis of all the other three states of consciousness. Given this conceptualization, the possibility of *Turiya* has important implications for theories of consciousness (Metzinger, 2019).

A variation of the concept of *Turiya* can be found in Kashmir Shaivism (Lakshmanjoo, 2015, 2017). According to this, there is a junction between each of three states, wakefulness, dreaming, and deep sleep. *Turiya* can be experienced in these junctions with practice. Some minor Upanishads and Kashmir Shaivism also propose a fifth state of consciousness called *Turyatita*, which is a state beyond *Turiya*. Kashmir Shaivism also talks of seven states of *Turiya* or bliss (Lakshmanjoo, 2017) in terms of progressive steps achieved through practice. These include *nijananda* (the bliss of your own self), *nirananda* (devoid of limited bliss), *prananda* (the bliss of breathing), *brahmānanda* (the bliss which is all-pervading), *mahananda* (the great bliss), *cidānanda* (the bliss of consciousness), and *jagadānanda* (universal bliss).

Buddhists also talk about different states or planes of consciousness. They mention four planes of consciousness, in which the fourth plane is called *Lokuttara*, which is unintentional consciousness (*nirvana*). *Nirvana* is a pure conscious state (Rao and Paranjpe, 2015). In later schools like *Vajrayana*, Buddha Nature (ultimate reality) is defined as permanence, bliss, purity, and self (Takasaki, 1966). The state of consciousness without content is problematic because such states are described as being non-intentional. Recent attempts to understand such states characterize non-dual consciousness in terms of multiple dimensions, which include presence or being, emptiness, non-representational reflexivity, bliss, luminosity, continuity, and singularity (Josipovic, 2019; Josipovic and Miskovic, 2020).

On a first pass, the definition of *Turiya* as given in the Upanishads or *samadhi* or *nirvana* seems formidable and difficult to capture by empirical methods. It is also possible that pure consciousness is conflated with absorption states like *samadhi* (Joispovic and Miskovic, 2020). So, the first question ignoring the difficulties posed by the definition is whether *Turiya* exists. If it does not exist (as defined?), then how do we understand the *Turiya* experience and how do we explain it? This paper will discuss one such prominent attempt, which is the ascending reticular arousal system (ARAS) model by Metzinger (2019).

If a state of consciousness without content (*Turiya* or *Nirvana*) does exist, then how do we study it? This paper sympathetically explores the possibility of consciousness without content and discusses possible ways to attack this problem. One possibility is to link it to states of consciousness or minimal phenomenal experience that are close in nature, study them, and interpolate (Baars, 2013; Windt, 2015). The second possibility is to argue for a need to postulate consciousness without content to explain specific properties of consciousness. In this paper, I will focus on the continuity of conscious experience and whether this necessitates postulation of consciousness without content, primarily based on Buddhist theories of consciousness. Finally, I will discuss current scientific theories in the context of consciousness without content.

## A Model of Minimal Phenomenal Experience (MPE)

The nature of consciousness and its phenomenal properties have also been investigated in western philosophy (Tye, 1997). For example, Metzinger (2019) quotes from Moore (1903) regarding transparency: “the moment we try to fix our attention upon consciousness and to see what, distinctly, it is, it seems to vanish: it seems as if we had before us a mere emptiness. When we try to introspect the sensation of blue, all we can see is the blue: the other element is as if it were diaphanous (Moore, 1903, p. 450).” The argument is that we can access only content but not content-carrying vehicle properties. Consciousness without content is not possible and consciousness is a second-order process. The second-order meta-awareness is generally not noticed but can be noticed through attention.

Based on phenomenological reports and analysis, Metzinger (2019, 2020) postulates certain phenomenological constraints for the minimal phenomenal experience (MPE). They are tonic alertness, absence of intentional content or content of “absence,” self-luminosity, introspective availability, epistemicity, and transparency. A state of full absorption is mostly characterized by wakefulness and self-luminosity. Lucid dreamless sleep is also somewhat similar to the state of full absorption, which is discussed in the next section.

Metzinger (2019) defines the minimal form of experience as: “constituted by the content of a predictive model serving to control and regulate the global signal of the ARAS, which in turn determines the brain’s general level of activation (pp. 1).” The model argues that this minimal phenomenal experience appears to be empty because it models a hidden cause of the ARAS signal, which is non-intentional vehicle property. The choice of the ARAS is due to its strength and its non-representational nature and this system needs to be controlled to obtain optimal level of arousal. While the ARAS signal itself is continuous, the ARAS model is discrete. In Metzinger (2020), the minimal phenomenal experience is defined in terms of a representation of tonic alertness maintained by the cingulo-opercular network (Sadaghiani and D’Esposito, 2015).

The model in essence argues that content-less consciousness is an illusion and the pure consciousness state actually has non-conceptual representational content. To be more specific, the model argues that the non-conceptual content is “empty” or “non-representational.” The “content-less” phenomenal state actually carries an abstract form of intentional content. Metzinger (2019, 2020) raises questions about taking the phenomenological reports as they are in terms of no-content. If it is the case that there is no sense of self or time, how could one remember that one was in such a state sans content or remember the duration or onset of such a state? In addition, it points to the fact that the experience and its report could be affected by the expectations and theories associated with such experiences in various contemplative traditions.

## States Close to Consciousness Without Content

Irrespective of whether the state of consciousness without content is actually without content or a special content (Metzinger, 2019), it is important to study such a state or reported experiences of such a state. Whether truly consciousness without content is possible or not, some have suggested focusing on states with minimal content as a way to get closer to reported experiences of such non-content states (Baars, 2013). Such suggestions include experiences based on repetition including Ganzfeld experiences and near threshold attending (Baars, 2013).

One possible way to study them would be to study neural measures associated with such a state with meditators who claim to experience such states (Hinterberger et al., 2014). In this electroencephalogram (EEG) study with experienced meditators, participants were asked to perform different types of meditation, which included thoughtless emptiness, focused attention, and open monitoring. Results showed that thoughtless emptiness is characterized not just by reduction in power of high frequencies but also low frequencies in EEG. In a more recent study (Winter et al., 2020) with a single experienced Buddhist meditator, conscious state without content was reported toward the end of the meditation session. EEG results showed a reduction in alpha power and increase in theta power during the self-reported content-free awareness period compared to the rest. The functional connectivity results showed decreases in the posterior default mode network and increases in connectivity in the dorsal anterior network. A direct comparison of the EEG results from the two studies show that the spectral analysis results do not confirm with each indicating the potential difficulties with studying such a state using EEG at present.

Some neural areas or systems have been proposed to underlie MPE states (Baars, 2013; Hinterberger et al., 2014; Josipovic, 2019). One proposal is the central precuneus network (Josipovic, 2014, 2019), which shows increased connectivity between central precuneus and the dorsolateral prefrontal cortex and could underlie nondual awareness. Another neural measure that has been proposed for minimal phenomenal experience is larger theta-alpha power perhaps accompanied by much reduced power in beta or gamma frequency ranges (Baars, 2013). Studies on sensory deprivation have also been used to study MPE states (Ben-Soussan et al., 2019) and insula has been proposed an important area for such states. However, there is very little conclusive evidence for neural mechanisms that underlie such states at the current juncture. It is also not clear whether these states have anything in common, which can also be discerned from the different neural substrates proposed in different studies (Baars, 2013; Hinterberger et al., 2014; Josipovic, 2014).

### Dreamless Sleep Experience

Windt (2015) proposes that “dreamless sleep experience can be described as pure temporal experience (pp. 35)” and could be considered a minimal phenomenal experience. In this state of dreamless sleep experience, there is experience of time but without any intentional content. The nature of dreamless sleep has been debated among different Indian philosophical systems (Thompson, 2014; Windt, 2015). Different Indian philosophical systems allow or disallow cognitive states without content. For example, the *Nyaya* does not allow objectless cognitive states but *Advaita* does. A state of consciousness without content or a pure temporal experience without content is possible according to *Advaita*. Given that pure self or consciousness is always present, *Advaita* argues that consciousness is present during dreamless sleep.

During such a dreamless sleep experience, the experience is that of a no-self and no intentional content. Windt (2015) argues that this experience can be understood as a phenomenal “now.” Using Husserl’s notion of retention (Husserl, 1991), Thompson (2015) argues that the recognition of absence of self and intentional content can possibly be based on retentional aspects of the “now.” While acknowledging, the possibility of dreamless sleep experience as a MPE, Metzinger (2019) points out potential issues with the characterization of dreamless sleep experience. These include contentlessness, atemporality, and epistemicity. The notion of an empty phenomenal now is not clear and phenomenology of the experience of consciousness without content is that of a lack of sense of time. In addition, Windt (2015) account does not take into account “passive, non-agentive knowing,” which captures the notion of “witnessing sleep.” These considerations pointed by Metzinger (2019) are critically important for not just dreamless sleep experience but also other MPE states, both in terms of similarities and differences.

## Continuity of Conscious Experience

One important debate about conscious perception is whether it is continuous or discrete (VanRullen and Koch, 2003; Dainton, 2014; Herzog et al., 2016; Fekete et al., 2018; White, 2018). It has been argued that conscious perception is discrete and the continuity of experience is as such an illusion (VanRullen and Koch, 2003; Herzog et al., 2016). Models of time perception, more specifically cinematic models assume discrete frames and imply that continuity of temporal experience is an illusion (Dainton, 2014).

Discrete models of perception generally assume that unconscious integrative processes occur over time and once the integration is complete, this results in all at once in conscious perception. This has been postulated to take around 100–500 ms. It has been argued that the conscious percept is an attractor in phase space (Herzog et al., 2016). Studies on attention have argued that attentional sampling is discrete (around 7–8 Hz) and this is a possible factor that underlies the discreteness of perception (VanRullen and Koch, 2003; VanRullen, 2016).

Arguments have been raised against the discrete model of mind or conscious perception (Spivey and Dale, 2006; Fekete et al., 2018; White, 2018). The proponents of continuous-time models of perception argue that the putative evidence for discrete perception is also consistent with continuous-time models of perception. Occasionally, the duration of the stimulus needed to consciously perceive a stimulus is conflated with the duration or timing of conscious experience (Thompson, 2014, p. 46–48).

White (2018) questions the boundaries of discrete temporal windows of momentary awareness, given that we already know that different perceptual modalities have different temporal resolutions. A gap of 30 ms may be required to segregate two flashes in foveal vision but a gap of 2–5 ms is enough to segregate two tones. In addition to temporal resolution differences across modalities, such differences also exist for different features within modalities. A classic example is the trade-off between peripheral and central vision in terms of spatio-temporal resolution. Moreover, integration of visual-auditory information themselves involve different timescales and our perceptual system can tolerate small asynchronies between the two (sounds and sight), still representing them as co-synchronous even with offsets around 50–100 ms to produce a unified audio-visual experience. White (2018) also considers the ability of such frames to explain feelings of flow, succession, and persistence of experiences beyond and within these frames. Would these problems be addressed by proposing a fourth state that is content less, non-representational, and continuous that underlie our experience?

The answer to the question of continuity (apparent or real) may have implications for the notion of consciousness without content. Different Indian systems argue for or against the continuity of consciousness (Waldron, 2003; Thompson, 2015). Many early Buddhist (Theravada and some Mahayana) theories argue for discrete moments of experience (Collins, 1982; Waldron, 2003; Thompson, 2015). However, for Buddhists, the discrete theories of consciousness do pose a problem in explaining other aspects of mind and consciousness. To quote from Evan Thompson, “How consciousness manages to function coherently, given that it is gappy. If consciousness is strictly momentary, in the sense that there is no consciousness whatsoever that persists during the gaps, then what accounts for its coherent functioning, not only from moment to moment but also across longer stretches of time? For example, what accounts for longer-lasting traits of consciousness, such as the attentional stability arising from meditation practice? Why do not the gaps between moments of awareness disrupt these continuities? (pp. 58).”

Different solutions have been proposed by different schools of Indian thought (Waldron, 2003; Thompson, 2014). The Theravada school distinguishes between active consciousness versus passive consciousness. Active consciousness is about the differing contents of experience. Here, passive consciousness is the basis of continuity of individual; “Life-continuum” or “factor of existence (*bhavanga*).” The passive exists only in the gaps between active (Waldron, 2003).

The Yogacara school argues for a underlying more base consciousness, which is continuously present at all time – *Alaya-vijnana* (store consciousness). The *alaya-vijnana* is the basis for cognitive awareness (which is probably discrete). This *alaya-vijnana* has no “I” or perspectivalness and it is the ego consciousness that brings in the “I (Waldron, 2003; Thompson, 2014).”

Sometimes *bhavanga* and *alaya-vijnana* have been interpreted as an unconscious base, which makes consciousness possible (Waldron, 2003; Rao and Paranjpe, 2015). If *bhavanga* or *alaya-vijnana* is interpreted as unconscious (but still presumably part of the mind) but continuous, then it is not clear what provides the continuity of conscious experience and it seems to simply move the problem of continuity of consciousness to continuity of non-consciousness. In addition, the term awareness or consciousness is explicitly used in many Buddhist texts in discussing *bhavanga* or *alaya-vijnana*. *Alaya-vijnana* is translated as store-house consciousness and need not be interpreted as an unconscious process (Kalupahana, 1992).

Generally, Buddhist theories of time assume time to be discrete (Collins, 1982; Waldron, 2003; Thompson, 2014). Theravada assumes that *bhavanga* itself is discrete and made of finer moments than consciousness with content. This stance implies that even *bhavanga* is gappy. It has been argued with consistent meditative practice that this momentariness may become perceivable. However, even if this is true then those who meditate should report a somewhat choppy consciousness without content experience. This is not usually reported even though loss of self and time are reported (Ataria et al., 2015).

Hierarchical theories of time perception assume time scales generally in the 30–100 ms range to a few seconds range (Pöppel, 1997). If *bhavanga* is made of moments and then is at a scale much smaller than 30 ms range, then these moments could be even of the order of less than 1 ms. From what we know of neuronal firings and their time scales, the discrete frames for a *bhavanga* would require neurons firing rates that would be difficult given their physical limitations. Of course, one can argue that *bhavanga* as fine discrete moments is not based on neuronal activations or new finer mechanisms would emerge but at this point there are no clear possible mechanisms available at such a fine temporal scale. The hierarchical nature of time perception itself can possibly achieved with nested, synchronized activity of populations of neurons oscillating at different frequencies, which are coupled and interact with each other (Roux and Uhlhaas, 2014).

Buddhist theories, in general, do use the metaphor of the stream of consciousness and especially describe *alaya-vijnana* as stream. Some have used *citta-santāna* or mind-stream as a synonym or alternative for *alaya-vijnana* (Lusthaus, 2013). For example, Kalupahana (1992) says “Instead of being a completely distinct category, *alaya-vijnana* merely represents the normal flow of the stream of consciousness uninterrupted by the appearance of reflective self-awareness. It is no more than the unbroken stream of consciousness called the life-process referred to by the Buddha. It is the cognitive process, containing both emotive and conative aspects of human experience, but without the enlarged egoistic emotions and dogmatic graspings characteristic of the next two transformations.”

Representational theories of consciousness like the global workspace theory (Baars, 2013) are generally not concerned with properties of conscious experience like continuity. The ARAS model postulated to handle MPEs is a special representational model and *prima facie*, it appears that is not concerned with explaining specific phenomenological aspects like continuity of conscious experience (Metzinger, 2019). In addition, while the ARAS signal is continuous, the ARAS model itself is not continuous.

## Consciousness Without Content and Theories of Consciousness

A prominent cognitive theory of consciousness is the global workspace theory (Baars, 2013). The global workspace theory, at its core, is a representational or functionalist theory. What one is conscious of is what is globally broadcasted in the brain or mind. If this is the case, and if consciousness is present without content, then this would imply that nothing is broadcast. This seems to go against global work space workspace theory and representationalist theories, in general (unless the no-content is made into a special non-intentional, non-conceptual content as in the ARAS model). Even if somehow workspace itself is represented and there is no other content, this would still be semantic content (Josipovic, 2019). The maintenance of any content in the global workspace would still need attention and monitoring.

While Baars (Baars, 2013; Josipovic and Baars, 2015) seems to be sympathetic to the possibility of consciousness without content, the implications of consciousness without content for global workspace needs to be explored in detail. It appears that *alaya*-*vijnana* or *bhavanga* awareness cannot be easily accommodated by purely content-based theories of consciousness, since processes operating on content are what makes cognitive or access consciousness possible.

How would other theories of consciousness address the possibility of consciousness without content? For example, consciousness has been conceptualized as a meaning-making process or producing information (Marchetti, 2018). Marchetti (2018) focusing on the content of conscious experience say that “the content of CI coincides with its form.” Given this conceptualization, it is not clear how consciousness without concept can be conceptualized. One could argue for the notion of “pure attention” as a process that does not have content but holds the system in a state of readiness within this theoretical framework (Marchetti, 2018). This is somewhat akin to the proposal of tonic alertness as a possible representational substrate for minimal phenomenal experience (Metzinger, 2020).

Integrated information theory (IIT) is another prominent theory that has been proposed to understand consciousness (Tononi, 2004; Tononi et al., 2016). Tononi et al. (2016) state “Similarly, IIT predicts that the cerebral cortex as a whole may support experience even if it is almost silent, a state which may perhaps be reached through meditative practices designed to achieve ‘naked awareness’ without content (pp. 460).” They also state “States of naked awareness could be compared with states of unawareness that occur, for example, during deep sleep or anesthesia, when the cause-effect repertoires of cortical neurons, regardless of the level of neuronal activity, are disrupted as a result of bistability (pp. 460).”

Dimensional models of consciousness (Berkovich-Ohana and Glicksohn, 2014; Paoletti and Ben-Soussan, 2019, 2020) also try to account for consciousness without content and how they can be achieved. In these dimensional models, time and emotion constitute two dimensions. The third dimension varies: access, varying from low accessibility to high accessibility (Berkovich-Ohana and Glicksohn, 2014) or motivation/self-determination (Paoletti and Ben-Soussan, 2020). The time axis goes from past to future and the emotion axis goes from reward to punishment. They intersect at a point which represent “present” in the time axis and zero emotion in the time axis. Defined in terms of access to awareness (Berkovich-Ohana and Glicksohn, 2014), the third axis goes from minimum access to maximum access or no-access to maximum access. In terms of self-determination (Paoletti and Ben-Soussan, 2019, 2020), the focus is on a particular form of intentionality to act and being aware. The origin or intersection of the all three dimensions possibly represents the state of consciousness without content, which they call the “place of pre-existence” (Paoletti and Ben-Soussan, 2019). It has been argued that such a state of no-self and lack of content is achieved through meditation or possibly sensory deprivation.

How would predictive processing theories handle consciousness without content? Some recent attempts have been made to understand meditation and meditative experience in the context of the predictive processing approach (Lutz et al., 2019; Pagnoni, 2019). Focused attention meditation can be conceived as a way to minimize prediction error through the processes of focusing attention and eliminating distractions with practice (Lutz et al., 2019). If we regard the mind as a hierarchical predictive control system (Jordan, 2003; Kumar and Srinivasan, 2012, 2014), then perhaps one is in a state of effortless perception in which prediction errors at all hierarchical levels are zero. This would include the ability to predict not signals from external environment but interoceptive signals from the body itself, which would need the ability to control the body as well. The ability to control both the body and mind is possible only through interactions with environment, which may partially address the dark room problem (Friston et al., 2012). If consciousness without content is possible, then it is not necessary to have a dark room *per se* to have absence of content in experience. If it is so, predictive processing theories may need to explain how it is that we have conscious experience, when there is no content (or minimal content) about which predictive inference needs to be made. Of course, it has been argued that the content is a special type of content, which gives rise to the phenomenological experience of no content (Metzinger, 2020). A speculative solution to this would be continuous-time models of perception, which can realize hierarchical predictive inference (Fekete et al., 2018) and may involve prediction of the vehicle (*bhavanga* or *alaya-vijnana*) alongside content of consciousness. That is predictive inference not just about the content of experience but also the dynamical structure of experience embedded possibly on a base consciousness.

One of the phenomenal aspects that is very rarely considered in most of these models or theories of consciousness, is *Ananda* or bliss. As discussed earlier, the Kashmir Shaivists talk of seven different states of bliss associated with *Turiya* (Lakshmanjoo, 2017). Since emotions or feelings are thought to be intentional mental states, it is not clear why there should be a reported experience of bliss, if there is no content. Consistent with this argument, bliss is not a phenomenal constraint for MPE according to Metzinger (2019). In the spherical models of consciousness (Berkovich-Ohana and Glicksohn, 2014; Paoletti and Ben-Soussan, 2019), the putative point in the three dimensional space representing a state of consciousness without content has zero emotion (neither pleasant nor unpleasant). It is not clear why this point is associated with reports of bliss. Proposers of nondual awareness do include bliss as one of the dimensions of such an awareness (Josipovic, 2019). The term *Brahman*, the underlying reality according to the Upanishads is generally characterized as *sacchidananda* (*sat* – existence or truth, *cit* – consciousness, and *ananda* – bliss). It could be important to consider how *ananda* is linked to consciousness without content or MPEs, in general.

## Conclusions

The presence or absence of content-less state of consciousness has important implications for theories of consciousness (Metzinger, 2019). Many current conceptions of consciousness do not consider a content-less state of consciousness as a possibility and would need to be significantly altered if such a state is possible. We need novel paradigms to study and theorize about such states of consciousness without content or minimal phenomenal experience. A thorough understanding of the phenomenal properties of consciousness and its links to functional or neurophysiological aspects would enable us build a comprehensive theory of consciousness (Josipovic and Miskovic, 2020; Metzinger, 2020). The current paper suggests that focusing on the continuity of conscious experience may necessitate proposing consciousness without content a theoretical necessity. Such states of consciousness have been reported for a long time among practitioners in various contemplative traditions and there is a need to take them seriously to eventually understand consciousness. It also seems to be the case that realizing such an experiential state seem to change one’s life in a significant manner. Hence there is also a need to measure the impact of having experienced such a state in day to day life of those practitioners.

## Author Contributions

The author confirms being the sole contributor of this work and has approved it for publication.

### Conflict of Interest

The author declares that the research was conducted in the absence of any commercial or financial relationships that could be construed as a potential conflict of interest.
